# Pharmacogenomics in oncology care

**DOI:** 10.3389/fgene.2014.00073

**Published:** 2014-04-08

**Authors:** Kelly K. Filipski, Leah E. Mechanic, Rochelle Long, Andrew N. Freedman

**Affiliations:** ^1^Epidemiology and Genomics Research Program, Division of Cancer Control and Population Sciences, National Cancer InstituteRockville, MD, USA; ^2^Pharmacological and Physiological Sciences Branch, Division of Pharmacology, Physiology, and Biological Chemistry, National Institute of General Medical SciencesBethesda, MD, USA

**Keywords:** pharmacogenomics, pharmacogenetics, somatic, germline, targeted therapy

## Abstract

Cancer pharmacogenomics have contributed a number of important discoveries to current cancer treatment, changing the paradigm of treatment decisions. Both somatic and germline mutations are utilized to better understand the underlying biology of cancer growth and treatment response. The level of evidence required to fully translate pharmacogenomic discoveries into the clinic has relied heavily on randomized control trials. In this review, the use of observational studies, as well as, the use of adaptive trials and next generation sequencing to develop the required level of evidence for clinical implementation are discussed.

## Introduction

In the last decade, the use of genomics in oncology significantly impacted treatment decisions for many patients. Oncologists have a variety of treatment options; however one patient may experience serious adverse events whereas another patient receives no therapeutic effect. Within a population, substantial variation exists resulting in unpredictable responses. Pharmacogenomics, the study of the interaction between the genome and clinical drug response (Monte et al., 2012), evaluates the associations between drug efficacy and toxicity and variation in drug metabolizing enzymes, receptors, transporters, and drug targets (Crews et al., 2012). The main priority of pharmacogenomics is to optimize treatment by understanding the underlying biological mechanisms and utilizing genomic contributions to treatment response to predict and individualize therapy and improve treatment outcomes. Unlike other diseases, cancer genetics must take into account both acquired (somatic) and inherited (germline) variation, both of which contribute to the efficacy and safety of a drug. However to date, integrated studies of germline and somatic variation have been limited. Somatic mutations are often attributed to treatment efficacy, whereas germline mutations are used to identify patients at highest risk of developing serious adverse events (Gillis et al., 2014). In this review, we will discuss examples of pharmacogenomic markers and differences in the evidence level required for implementation into clinical care. In addition, several mechanisms for developing evidence for clinical implementation and new technologies entering oncology practice will be discussed.

## Somatic mutations and targeted therapy

Somatic mutations have highlighted the importance of understanding the underlying biology of cancer with discoveries elucidating the primary genetic changes driving tumorigenesis providing molecular drug targets. Prospective tumor sequencing is being increasingly utilized, changing the paradigm of cancer treatment from site specific cytotoxic treatment, to molecularly targeted treatment (MacConaill et al., 2011; Ong et al., 2012). Many drugs are being developed for defined molecular targets (Simon, 2013), one such example is the use of crizotinib in anaplastic lymphoma kinase (ALK) positive non-small cell lung cancer (NSCLC). Tumor DNA sequencing identified two patients with NSCLC harboring novel *ALK* rearrangements as crizotinib, a tyrosine kinase inhibitor, moved into clinical trials. Both patients showed marked response to crizotinib prompting protocol amendments to prospectively test for *ALK* rearrangements throughout clinical development (Ou, 2011; Ou et al., 2012). The fortuitous discovery of *ALK* rearrangements during phase I trials of crizotinib restricted development to a subset of patients relying heavily on rigorous randomized controlled trials with an appropriate companion diagnostic to select patients. Predictive testing for biomarkers like *ALK* reduces unnecessary treatment in patients that will not respond and helps avoid potentially toxic effects of treatment (Ong et al., 2012). Molecularly targeted therapies like crizotinib have replaced cytotoxic therapy as standard of care in several cancer types including breast cancer, NSCLC, and melanoma (Ong et al., 2012; Gillis et al., 2014). Randomized clinical trials (RCTs) have been important to modern medicine, however the shift away from average treatment effects within a whole population to molecularly defined sub-populations is new to clinical trial design, and will be discussed later in this review.

## Germline mutations

In oncology, germline mutations play a significant role in the treatment response to both chemotherapy and targeted anti-cancer agents. These mutations are often associated with the pharmacokinetics of a drug contributing to treatment related adverse events experienced by patients (Hertz and McLeod, 2013; Gillis et al., 2014). In this regard, germline pharmacogenomic markers can identify patients at highest risk of developing serious adverse events that could subsequently lead to treatment discontinuation and failure like musculoskeletal pain after treatment with aromatase inhibitors. Severe musculoskeletal pain has been reported in up to half of women treated with aromatase inhibitors contributing to a treatment discontinuation rate of about 10% (Crew et al., 2007; Henry et al., 2008; Ingle et al., 2010). Ingle et al. found four single nucleotide polymorphisms (SNPs) mapping to the T-cell leukemia 1A (*TCL1A*) gene were associated with the development of musculoskeletal adverse events in patients receiving adjuvant aromatase inhibitors (Ingle et al., 2010). Subsequent functional studies revealed that *TCL1A* was induced by estrogen with higher levels of expression in cells with the variant alleles for these SNPs. Further results suggested an estrogen dependent, *TCL1A* SNP-dependent regulation of cytokines, cytokine receptors, and NF-κB transcriptional activity. These SNP-dependent changes may help to elucidate the pathway involved in musculoskeletal pain following aromatase inhibitor mediated estrogen deprivation (Liu et al., 2012). The strategy of discovering genetic variants and studying the underlying biology of the association is central in pharmacogenomic studies. It outlines a strong biological basis for the genetic association and provides mechanistic insight into the biology of the event that could lead to new drug targets to prevent the toxicity. Pharmacogenomic markers like *TCL1A* are extremely important when taken into context with not only the large number of women that could be exposed to aromatase inhibitors, but the fact that many of those women will have long term survival after receiving aromatase inhibitors and may experience decreased quality of life due to musculoskeletal pain. However, like many pharmacogenomic markers, *TCL1A* may never be used in clinical practice because a large randomized clinical trial will never be completed to study the association, even though other treatment options are available and with the understanding of the biology prevention strategies could be developed.

In addition to adverse events and pharmacokinetics of a drug, germline mutations may influence drug efficacy. Recently a germline mutation in the proapoptotic gene *BIM* was associated with the resistance to tyrosine kinase inhibitors in chronic myeloid leukemia (CML) and epidermal growth factor receptor (EGFR) mutant NSCLC. Identification of this mutation not only explains some of the poor response seen in patients with CML treated with imatinib, but also provides biological insight into different strategies to overcome the resistance that are currently in preclinical testing (Cheng and Sawyers, 2012; Ng et al., 2012). Although still in development, *BIM* is an important reminder that only focusing on somatic or germline variation investigators can miss key mutations that affect treatment outcomes.

One of the most well known pharmacogenomic markers is the association of thiopurine-S-methyltransferase (TPMT) and mercaptopurine (6-MP). Mercaptopurine is an important component of pediatric acute lymphoblastic leukemia (ALL) treatment, and is used in the treatment of some nonmalignant diseases (Paugh et al., 2011). A variant in the *TPMT* gene reduces the function of the enzyme leading to excessive levels of cytotoxic thioguanine nucleotides (6-TGNs) subsequently leading to an increased risk of severe myelosuppression (Paugh et al., 2011). Although a randomized clinical trial has never been done, the Food and Drug Administration (FDA) agreed that the evidence was sufficient to mention testing for TPMT deficiency, thus allowing identification of safe doses of mercaptopurine without compromising efficacy (Relling et al., 2011). Ample evidence, including *in vitro* and retrospective analyses provide support for the use of *TPMT* testing in patients receiving mercaptopurine to prevent serious treatment induced myelosuppression (Relling et al., 2011), but the consistent and widespread use of pre-treatment *TPMT* testing has not been universally accepted.

## Strategies for developing pharmacogenomic evidence

The field of pharmacogenomics has uncovered an abundance of actionable and clinically relevant markers including both somatic (Table 1) and germline (Table 2) mutations. Prospective screening for predictive pharmacogenomic markers, like TPMT, may enhance treatment response by reducing the risk of toxicity from systemic drug concentrations while maintaining the anti-cancer activity of the drug. However, implementation into clinical practice has been slow, in part due to contradictory professional guidelines and recommendations, and differing thresholds for evidence (Gillis et al., 2014). RCTs have been the gold standard for evidence in treatment response and genetic testing and for pharmacogenomic markers like ALK that were discovered during the drug development process RCTs are feasible. Randomization in experimental studies attempts to control for biases by balancing factors that affect outcomes across study groups. However, clinical trials are not always the most feasible approach for developing evidence due to cost, time constraints, and the large sample size necessary to complete a trial (Gillis et al., 2014). Observational studies are more prone to a number of biases and the potential confounding which may lead to incorrect results. But these studies offer some advantages over clinical trials including larger numbers of subjects at an affordable cost, ability to examine meaningful genomic subgroups, longer follow-up times, and ability to examine drugs and their interactions with the genome that are used off-label (Dreyer et al., 2010). Rigorously designed high-quality observational studies can play a particularly important role in developing evidence for decision making in cancer pharmacogenomics because they can relatively quickly generate results that are applicable to real-world situations and examine long-term risk and benefits. Below we discuss the use of epidemiologic studies for developing evidence to support the use of pharmacogenomic tests where RCTs are not an option.

**Table 1:** **Selected somatic pharmacogenomic markers**.

**Pharmacogenomic marker**	**Drug (s)**	**Genome**	**Outcome**	Multi-tumor marker^*^
ABL	Bosutinib, dasatinib, imatinib, nilotinib, ponatinib	Somatic	Efficacy	Yes
ALK	Crizotinib	Somatic	Efficacy	Yes
BRAF	Vemurafenib	Somatic	Efficacy	Yes
EGFR	Afatinib, cetuximab, erlotinib, panitumumab, vandetinib	Somatic	Efficacy	Yes
FcγR	Cetuximab, rituximab, trastuzumab	Somatic	Efficacy	No
HER2	Lapatinib, pertuzumab, trastuzumab, trastuzumab emtansine	Somatic	Efficacy	No
KRAS	Cetuximab, panitumumab	Somatic	Efficacy	Yes
KIT	Imatinib	Somatic	Efficacy	Yes
MET	Trametinib	Somatic	Efficacy	Yes

* Commercially available multi-marker tumor panels.

Arup Laboratories (http://ltd.aruplab.com/Tests/Pub/2007991).

AsuraGen (http://asuragen.com/products-and-services/genomic-services/next-generation-sequencing-services/).

Foundation Medicine (http://www.foundationone.com/).

**Table 2:** **Selected germline pharmacogenomic markers**.

**Pharmacogenomic marker**	**Drug (s)**	**Genome**	**Outcome**	Multi-tumor marker^*^
BIM	Imatinib	Germline	Efficacy	No
CYP2B6	Cyclophosphamide	Germline	Toxicity	No
CYP2D6	Tamoxifen	Germline	Efficacy	No
DPYD	Capecitabine, fluorouracil	Germline	Toxicity	No
G6PD	Rasburicase	Germline	Toxicity	No
MLH1, MSH2, MSH6, PMS2	Fluorouracil	Germline	Efficacy	Yes
SLCO1B1	Methotrexate	Germline	Toxicity	No
SLC28A3	Anthracyclines	Germline	Toxicity	No
TCL1A	Aromatase inhibitors	Germline	Toxicity	No
TPMT	Mercaptopurine, thioguanine, cisplatin	Germline	Toxicity	No
UGT1A1	Irinotecan	Germline	Toxicity	No

* Commercially available multi-marker tumor panels.

Arup Laboratories (http://ltd.aruplab.com/Tests/Pub/2007991).

AsuraGen (http://asuragen.com/products-and-services/genomic-services/next-generation-sequencing-services/).

Foundation Medicine (http://www.foundationone.com/).

### Retrospective observational study

Leveraging clinical trial data in a retrospective case-control study, where all the patients are receiving treatment, the cases and controls are those affected and unaffected by the outcome of interest, is a powerful design approach for detecting common variants associated with phenotypic traits (Ritchie, 2012). These retrospective analyses have proven successful for both somatic and germline mutations. Cetuximab, an anti-EGFR monoclonal antibody is used in the treatment of colorectal cancer, however only a subset of patients responded to treatment. Tumor samples from cetuximab treated patients were retrospectively analyzed identifying mutations in the *KRAS* gene that were associated with response to therapy (Karapetis et al., 2008; Van Cutsem et al., 2009). Patients that are mutation positive do not respond to cetuximab, leading to the development of a companion diagnostic for *KRAS* testing and changes to the prescribing information for cetuximab to only be used in *KRAS* mutation negative patients. Although only retrospective analyses were performed, professional societies and the FDA recommend *KRAS* testing prior to initiation of cetuximab therapy (Kelley et al., 2011; Evaluation of Genomic Applications in Practice and Prevention Working Group, 2013).

### Electronic medical records

The electronic medical record (EMR) provides a wealth of information to study questions at the population level. Large patient cohorts can be quickly created for a variety of diseases and drug response phenotypes to better evaluate drug safety. With the establishment of biobanks concurrent with the adoption of EMR, studies of genomic associations of drug response may become more comprehensive. The NHGRI funded Electronic Medical Records and Genomics (eMERGE) Network is leading the way by developing best practices for EMR guided genomic studies (Gottesman et al., 2013). These population based studies have access to much larger populations of patients making it easier to study rare, but serious, adverse events. One eMERGE site, Marshfield Clinic Personalized Medicine Project (PMRP), identified genetic factors associated with thromboembolic events in women taking tamoxifen. Patients were identified for a study by interrogating the EMR for breast cancer diagnosis, tamoxifen treatment, and thromboembolic event. SNPs in the *ESR1* gene were shown to be associated with thromboembolic events in women treated with tamoxifen. These events are relatively rare (1.7–8.4%) therefore using this large practice based cohort, investigators were able to capture enough events to evaluate the underlying genetic difference in women with and without thromboembolic events (Onitilo et al., 2009).

EMR based studies provide some advantages over RCTs in evaluating drug safety in the general population. Biobanks of several sites can participate together in investigations, thus increasing sample size and study power. In addition, clinical trials exclude patients with comorbidities, taking concomitant medications, and may include run in periods excluding patients that show drug intolerance prior to randomization. In the unrestricted EMR populations a higher incidence of adverse events may be identified (Wilke et al., 2011).

### Emerging technologies and trial design

In addition to retrospective analyses and EMR studies to develop evidence for the use of pharmacogenomic tests in clinical practice, the acceptance of new technologies and clinical trial designs are imperative to improving clinical outcomes. Both the time and cost of genotyping has been steadily decreasing allowing for the creation of denser genotyping panels and advent of next generation sequencing (NGS) has allowed for a more complete view of the genome. There are three types of NGS: whole exome sequencing, whole genome sequencing, and targeted gene panels (Rehm et al., 2013). Unlike genotyping of candidate genes and genome wide association studies, WGS can identify rare variants that may be very important in the genomic contribution to treatment response and toxicity (Gillis et al., 2014), particularly for a drug like methotrexate that has wide inter-individual differences. A GWAS identified SNPs annotated to the transporter gene, *SLCO1B1* that were associated with methotrexate clearance (Trevino et al., 2009). Deep resequencing of the gene was done and Ramsey et al. found that rare variants, only identified through deep sequencing accounted for 17.8% of the genes effect on clearance (Ramsey et al., 2012). NGS can provide not only common variants, but a comprehensive catalog of rare variants that may have a larger effect size than common variants contributing to the overall phenotype of drug response (Gillis et al., 2014). There are some challenges with NGS, particularly the daunting task of handling and interpreting NGS results.

With the advent of molecularly targeted therapies, prospective use of NGS is being incorporated into adaptive clinical trials, using the genomic landscape of the tumor to direct therapy as opposed to site specific treatment (MacConaill et al., 2011; Ong et al., 2012). Trials such as the National Cancer Institute's MPACT trial are prospectively evaluating tumor mutations for actionable variants and then randomizing patients to receive a drug targeting the aberrant pathway or standard of care regardless of tumor site (Simon and Polley, 2013). Forward thinking trials like MPACT are poised to change the way tumors are classified and patients are treated. Already targeted gene panels have emerged focused on a limited set of genes creating a greater depth of coverage and improving the ability to interpret the findings in the clinic (Rehm et al., 2013). Commercially available multi-tumor marker panels are primarily focused on somatic mutations with the inclusion of several known driver mutations predictive of treatment response to molecularly targeted therapies (Table 1), however to date, it is unknown how comprehensive these panels are, whether they are including the optimal set of genes, and the broader utility of these panels has yet to be evaluated. There are challenges and limitations to the use of NGS, but NGS and multi-marker tumor panels may provide an opportunity to generate incredible amounts of information about the tumor genome and thus drive research to elucidate some of the biological mechanisms underlying the tumor growth and survival.

## Conclusion

Pharmacogenomics has provided significant impact in oncology treatment, but implementation differs between somatic and germline markers. Developing sufficient evidence for use of pharmacogenomic markers has been difficult, in part because RCTs cannot be performed for every marker identified. A move toward retrospective analyses, large population studies using EMRs, and the establishment of mechanism-based evidence can propel progress in the field. In addition, embracing new methods, such as NGS and adaptive clinical trials, is providing a wealth of information about tumor biology and changing the landscape of cancer treatment. To date, pharmacogenomic studies tend to focus on either somatic or germline mutations in isolation from each other, but to truly optimize clinical care both genomes need to be integrated into clinical decision making. Both well designed, forward thinking trials and population based studies are necessary to fully implement pharmacogenomics into clinical care.

### Conflict of interest statement

The authors declare that the research was conducted in the absence of any commercial or financial relationships that could be construed as a potential conflict of interest.
